# Updates on the diagnosis and monitoring of giant cell arteritis

**DOI:** 10.3389/fmed.2023.1125141

**Published:** 2023-02-23

**Authors:** Sara Monti, Valentin Sebastian Schäfer, Francesco Muratore, Carlo Salvarani, Carlomaurizio Montecucco, Raashid Luqmani

**Affiliations:** ^1^Department of Internal Medicine and Therapeutics, Università di Pavia, Pavia, Italy; ^2^Division of Rheumatology, Fondazione IRCCS Policlinico San Matteo, Pavia, Italy; ^3^Clinic of Internal Medicine III, Department of Oncology, Hematology, Rheumatology and Clinical Immunology, University Hospital of Bonn, Bonn, Germany; ^4^Rheumatology Unit, Azienda USL-IRCCS di Reggio Emilia, University of Modena and Reggio Emilia, Reggio Emilia, Italy; ^5^Rheumatology Department, Nuffield Orthopaedic Centre, University of Oxford, Oxford, United Kingdom

**Keywords:** giant cell arteritis, diagnosis, monitoring, imaging, biopsy

## Abstract

This mini-review offers a critical appraisal of the currently employed imaging or histopathological tools to diagnose and monitor giant cell arteritis (GCA). An overview of the most updated evidence and current application of color duplex ultrasonography (US), temporal artery biopsy (TAB), 18-fluorodeoxyglucose [18F] FDG-PET/CT, magnetic resonance imaging, and computed tomography angiography is provided. The main limitations of each tool, and the most relevant research developments are discussed. The review highlights the complementary value of the available modalities to ensure a correct diagnosis of GCA, and to provide valuable prognostic information. Novel evidence is accumulating to support the role of imaging, and particularly US, as a monitoring tool for the disease, opening new perspectives for the future management of large vessel vasculitis.

## 1. Introduction

In recent years, the management of giant cell arteritis (GCA) has been going through some paradigmatic changes. Even though the first report of the potential applicability of color duplex ultrasonography (US) for the diagnosis of GCA dates back to 1997 with the first description of the “halo sign” as an indication of inflammatory vessel wall edema (1), it was only in 2018 that formal international consensus was achieved (2) and dedicated recommendations for the use of imaging in large vessel vasculitis (LVV) became available (3). Temporal artery biopsy (TAB) remains the gold standard for the diagnosis of GCA with optimal specificity, however, recent studies have proven a higher diagnostic yield, cost-effectiveness, and prognostic impact of imaging (4, 5). Indeed, the introduction of fast-track clinics for the urgent referral of patients with suspected GCA to be assessed clinically and with US has significantly reduced the rate of permanent visual loss for these patients compared to standard clinical practice (5–7). Moreover, increasing knowledge of the clinical characteristics and outcomes of large-vessel GCA (LV-GCA) have shed new light on the importance of assessing extra-cranial involvement in patients with GCA (8, 9). Moreover, the use of imaging as a monitoring tool for LVV has long been affected by uncertainties regarding the exact meaning of residual subclinical inflammatory findings in patients in remission. Nevertheless, new evidence is accumulating to support a potential role for imaging, and especially US, as a monitoring tool to assess response to treatment and detect relapses in patients with GCA (10). Finally, the assessment of biologic drugs in randomized controlled trials of GCA has significantly improved the therapeutic options for these patients and has provided new flourishing research in the field (11).

## 2. Updates on the use of temporal artery biopsy for giant cell arteritis

A definite diagnosis of GCA often requires a TAB (12). TAB is a mini-invasive procedure with low risk of complications, generally performed under local anesthesia on an outpatient basis. Both EULAR and ACR recommend unilateral TAB or temporal arteries imaging in all patients presenting with symptoms compatible with GCA, in particular in those with cranial manifestations (13, 14). US of the temporal arteries has shown good sensitivity and specificity for the diagnosis of GCA when performed by operators with expertise in the technique, and, in these circumstances, it can be considered a diagnostic surrogate for TAB. However, in the centers without long-standing expertise in temporal artery US, and in all cases in which temporal artery US is negative in a clinically suggestive case, TAB remains the recommended diagnostic test for the diagnosis of GCA(13, 14).

The classic histologic picture of GCA is a transmural inflammatory infiltrate consisting of lymphocytes, macrophages, and, in approximately 75% of cases, giant cells. The lesion frequently has a “concentric rings” appearance, with a thicker inflammatory band surrounding the external elastic lamina and a thinner inflammatory band along the internal elastic lamina. A peculiar laminar necrosis, consisting of a band of acellular eosinophilic material sometimes bordered by palisading histiocytes along the internal elastic lamina, is present in approximately 25% of cases. Fibrinoid necrosis is extremely rare, and its presence should prompt consideration for the possibility of an alternative diagnosis (i.e., one of the systemic necrotizing vasculitides). In around 20% of positive TABs, the inflammatory infiltrate (typically lymphocytic) is restricted to the adventitial vasa vasorum or periadventitial small vessels (15). Most of these patients have a final diagnosis of GCA (16), even if the presence of restricted inflammation at TAB has low sensitivity and specificity for GCA diagnosis. To date, the diagnostic and prognostic significance of these restricted forms of inflammation remains unknown and in these cases, GCA diagnosis and treatment should be based on clinical ground (17).

In the absence of a definitive diagnostic test for GCA, it is hard to estimate the diagnostic performance of TAB for the diagnosis of the disease. The specificity of TAB is excellent, approaching 100%, but the most important limitation of TAB remains the lower sensitivity, that ranges from 50 to 95% in most studies (18). A recent systematic literature review and meta-analysis provided a pooled sensitivity of 77.3% (95% CI: 71.8, 81.9%) of TAB for the diagnosis of GCA, showing indirect evidence that TAB is not less sensitive than temporal artery imaging for the diagnosis of GCA (18). Expertise is important also in the pathologist’s ability to evaluate TAB and discern which features are compatible with GCA. In a multicenter study in which pathologists were not trained in the evaluation of TABs, the sensitivity of TAB for the diagnosis of GCA was 39%, significantly lower than that reported in previous studies in which a single pathologist expert in GCA reviewed all TABs (4, 18, 19).

The sensitivity of TAB for the diagnosis of GCA may also be affected by:

- *Biopsy length, number of sections evaluated and bilaterality of the procedure:* False-negative biopsies are usually attributed to the patchy involvement of the temporal artery, where areas of inflamed artery may alternate with areas of normal artery (skip lesions). In order to minimize the risk of skip lesions, and thus of a false negative result, it is generally recommended to remove longer segments of temporal artery. However, a post-fixation TAB specimen longer than 5 mm may suffice to reduce the risk of a false negative result according to two recent studies that retrospectively evaluated 1,520 and 694 TABs, respectively. Nevertheless, international recommendations still suggest that a long-segment temporal artery biopsy (>1 cm) should be preferred (14). Since the arterial specimen shrinks after excision, surgeons should remove a temporal artery segment longer than 10 mm to improve the diagnostic yield of TAB (20, 21). Furthermore, inflamed sections are found at deeper levels in 6–12% of TABs in which the first section was uninflamed (21, 22). In all TABs showing a negative first section, at least three additional deeper biopsy sections should be cut and evaluated by the pathologist to reduce the risk of a false negative result. The increased yield of contralateral biopsy for the diagnosis of GCA is in the range of 5% (23). Unilateral biopsy, possibly from the symptomatic side, is recommended. Contralateral biopsy is suggested only in cases of a first negative or inappropriate result and high clinical suspicion of cranial GCA (14).
- *Glucocorticoid treatment:* The inflammatory infiltrate involving the wall of the temporal arteries resolves slowly after starting glucocorticoid treatment, persisting for at least 2–4 weeks (24–26). Recommendations suggest to ideally obtain TAB within 2 weeks as the sensitivity decreases from 78% (within 2 weeks) to 65% (within 2–4 weeks) (14). However, inflammatory changes indicating GCA may still be present after 4 or more weeks of glucocorticoid treatment. A longitudinal histopathologic study reported that GCA may still be demonstrated on repeated TABs in 75% at 6 months, and 44% at 12 months (14). In order to maximize the diagnostic yield of the procedures, TAB should be obtained within 2–4 weeks after starting glucocorticoid therapy. Beyond this time limit, TAB may be considered in selected cases at the discretion of the physician and the patient (27). The role of TAB as a monitoring tool for treatment response has been previously reported in a limited number of patients by gene expression analysis, showing decreased pro-inflammatory activity and increased vascular remodeling (28).
- *Disease phenotype:* Extra-cranial or large vessel GCA indicates the inflammatory involvement of the aorta and its major branches. These patients typically lack cranial manifestations and are often asymptomatic or can present with systemic manifestations and refractory polymyalgic symptoms. When performed in patients with suspected GCA, TABs are positive in 25–35% of cases, mainly in patients with the cranial phenotype of the disease, and inadequate in around 4% (14). US-guided TAB does not improve the sensitivity of TAB for diagnosing GCA, but US may be useful for locating the artery before or during the biopsy procedure, reducing the risk of inadequate specimens (9).

In clinical practice, TABs performed for evaluation of patients with suspected GCA are positive in 25–35% of cases, and inadequate in around 4% (21). US-guided TAB does not increase the positive yield of TAB but is useful for locating the artery in preparation for the biopsy procedure, reducing the proportion of inadequate specimens (29).

## 3. Updates on the use of ultrasound for giant cell arteritis

The current international EULAR recommendations indicate US as the preferred early imaging test in patients with a suspected clinical diagnosis of GCA. Moreover, in patients with a high clinical probability for the diagnosis, and a supportive imaging test, the diagnosis can be confirmed without the need for further testing. US of the temporal and/or axillary arteries should be the primary imaging test in patients with predominantly cranial features provided that adequate expertise and equipment are available (3). The halo sign has been recently defined by an Outcome Measures in Rheumatology (OMERACT) working group as a “homogenous, hypoechoic wall thickening that is well delineated towards the luminal side and is visible both in longitudinal and transverse planes, most commonly concentric in transverse scans” and represents the main US finding in active GCA (2). The compression sign is used to confirm the presence of a halo and is less dependent on the operator’s experience (2). Nonetheless, high expertise and adequate equipment, including a high frequency probe (>15 MHz) are essential to ensure reliable temporal artery exploration with good sensitivity. Previous evidence informing EULAR recommendations had provided a pooled sensitivity for the halo sign of 77% (95% CI: 62–87%) and pooled specificity of 96% (95% CI: 85–99%) compared with a clinical diagnosis of GCA (30). The most recent systematic literature review and meta-analysis has confirmed the good sensitivity [67% (95% CI: 51, 80)] and specificity [95% (95% CI: 89, 98%)] of the halo sign in the diagnosis of GCA (Table 1) (31). Overall, US has a significantly better sensitivity than TAB while retaining very high specificity, reaching 100% in case of bilateral halo. Moreover, US can easily be implemented as a point-of-care test in dedicated fast-track clinics for the early diagnosis of GCA. Fast-track clinics are currently available in a growing number of specialist referral centers for the care of patients with LVV, leading to a substantial reduction in the rate of permanent blindness (6, 7). Nevertheless, the relapse rate during follow-up did not seem to be reduced since the introduction of fast-track clinics (5), highlighting the unmet need of appropriate risk stratification and tailored treatment based on the clinical characteristics of GCA at diagnosis. The core US assessment of GCA provides the best diagnostic yield balanced with the time needed to perform the procedure and includes scanning of the temporal arteries along the whole length of their common, parietal, and frontal branches bilaterally, and the axillary arteries (32). Several studies, including some recent evidence, have assessed the adjunctive role of extended US protocols including the assessment of other cranial or extra-cranial arteries confirming the generally optimal sensitivity and specificity of the core set (temporal and axillary arteries). In a recent study including 83 patients with GCA, the inclusion of the subclavian artery increased the sensitivity by 1%, and the inclusion of the brachiocephalic and common carotid arteries increased the sensitivity by 3% (33). Nevertheless, the deep anatomical distribution and difficulties in examination make the assessment of the brachiocephalic artery trunk subject to variation and lack of reproducibility. Generally, besides research purposes, the extension to other explorable vessels can be suggested in patients with a high clinical probability of GCA in whom the temporal and axillary arteries do not display signs of active GCA.

**TABLE 1:** Diagnostic performance and monitoring utility of the different imaging tools available for the assessment of giant cell arteritis.

	Sensitivity	Specificity	Role in monitoring the disease
TAB (4, 17)	Ranges 39–77.3% (95% CI: 33, 81.9)	100% (95% CI: 97, 100)	Invasive procedure limits repeatability in clinical practice. Vasculitis still demonstrated on repeated biopsies up to 12 months from diagnosis; tissue pro-inflammatory markers change in response to treatment.
Ultrasound (30, 31)	Ranges 54–81% (95% CI: 48, 88) compared with a clinical diagnosis of GCA Ranges 70 (95% CI: 56, 81) compared to TAB	Ranges 95–96% (95% CI: 85, 99) compared with a clinical diagnosis of GCA Ranges 84 (95% CI: 73, 91) compared to TAB	Significant sensitivity to change of halo count and intima-media thickness in response to treatment. US findings of temporal artery correlate with signs of disease activity. Emerging role in detecting relapses.
MRI (30, 57, 64)	Ranges 73–75% (95% CI: 57, 85) compared with a clinical diagnosis of GCA Ranges 91–93% (95% CI: 89, 96) compared to TAB 78.4% for cranial MRI	Ranges 88–89% (95% CI: 81, 92) compared with a clinical diagnosis of GCA Ranges 78–81% (95% CI: 73, 87) compared to TAB 90.4% for cranial MRI	Monitoring role of cranial MRI is being currently investigated. Reduced findings after 5 days of high-dose glucocorticoids. Persistent vessel wall enhancement described in extra-cranial arteries in one third of patients in remission treated with tocilizumab.
PET (30, 43, 65)	Ranges 61–80%; 73.3% for cranial arteries 77% compared with a clinical diagnosis of GCA 67% compared to TAB	Ranges 66–100%; 97.2% for cranial arteries 100% compared with a clinical diagnosis of GCA 66% compared to TAB	Controversy on the significance of a persistent uptake in patients in clinical remission (vascular remodeling? Subclinical activity?). PET vascular activity score (PETVAS) has been used to assess response to treatment.
CTA (30)	73% (95% CI: 45, 92) for a diagnosis of LV-GCA	78% (95% CI: 40, 97)	Useful for the long-term monitoring of structural damage (aneurysms/stenosis).

While the accepted definition for a diagnostic US in GCA is based on qualitative ultrasonographic findings and halo compressibility, and a definite consensus has not been reached, studies have identified cut-off values for the intima media thickness (IMT) that can distinguish vasculitic from normal arteries (34, 35). A normal temporal artery in a 70 years old patient has an IMT of ∼ 0.2 mm, while an inflamed artery has an IMT of ∼ 0.5–0.6 mm; a normal axillary artery has an IMT of ∼ 0.6 mm, while an inflamed artery in a patient with GCA has an average IMT of ∼ 1.7 mm. The proposed cut-off values range between 0.29 and 0.42 mm for the different branches of the temporal artery, and 1.0 mm for the axillary arteries (35). Similar cut-off values with high levels of diagnostic accuracy (≥0.4 mm for temporal, facial and occipital arteries, ≥0.7 mm for vertebral arteries, and ≥1 mm for carotid, subclavian and axillary arteries have been proposed by other research groups (34).

Ultrasonography has traditionally been considered in a binary fashion (positive/negative according to the presence of a halo in at least one of the assessed vascular territories), however, recent research trends have focused on the role of a quantitative assessment of US findings combining information on the number of sites with halos and the degree of the IMT measurable by US (36). The disease extent and severity as measured by US quantitative scores has been demonstrated to have important diagnostic value, and has been correlated with the probability of having a diagnostic TAB (36). Moreover, quantitative US scores have been associated with the probability of ocular ischemia at diagnosis (37). On the other hand, the prognostic role of a baseline quantitative score over follow-up is still to be defined (36).

The increasing interest in the quantitative US findings in GCA has led to a better understanding of the halo characteristics in response to treatment and has provided important evidence on the monitoring potential of this tool (Table 1). IMT size in the temporal arteries (but not in the axillary arteries) has been demonstrated to reduce following the first 7 days of glucocorticoid treatment supporting its role as an early marker of disease activity (38). Moreover, sensitivity to change in response to treatment has been demonstrated for the halo sign (in terms of number of halos and IMT thickness) starting from week 1 throughout week 24 for the temporal artery, and only after week 6 for the axillary halo features. Moreover, the number of temporal artery segments with halo and maximum halo IMT show significant correlation with signs of disease activity (erythrocyte sedimentation rate, c-reactive protein, Birmingham Vasculitis Activity Score) and cumulative glucocorticoid doses. On the other hand, halo at the level of the axillary arteries seems to display a different behavior without significant correlation with other aspects of disease activity (10).

Quantitative US has been employed in a randomized controlled trial to monitor the response to treatment to high-dose glucocorticoids and Tocilizumab, demonstrating the remission-induction effect of Tocilizumab and supporting the important monitoring role of US (39).

The monitoring utility of US has also been demonstrated by the ability to effectively detect relapses. Halo sign has been identified in 94% of first disease relapses in an international cohort of patients with GCA followed with a standardized protocol, but with a lower mean number of segments with halo and sum of halo IMT compared to disease onset (10, 32).

The monitoring assessment of GCA with US has provided valuable information not only on the quantitative changes of the halo over time, but also on the qualitative modification of halo, particularly for chronic changes at the level of the axillary arteries. The OMERACT definition and reliability assessment of chronic US lesions of the axillary artery has been provided for patients with long-standing GCA. The definition is based on measurement and appearance of the intima media complex. The inter- and intra-reader reliability of the new definition among experts was good to excellent (40). Moreover, the IMT of the axillary arteries is known to decline more slowly than the temporal artery, with a reduction persisting in the first 18 months of treatment. An IMT of 0.87 mm has been proposed to be highly specific (specificity 96%, sensitivity 61%) for the diagnosis of chronic axillary involvement in GCA (41).

## 4. Current use and new aspects regarding other imaging modalities (other than US)

### 4.1. 18-fluorodeoxyglucose FDG-PET/CT

FDG-PET/CT has proven to be highly accurate in identifying large vessel GCA. Several studies have looked at its diagnostic performance and determined that it has a sensitivity of 61–80% and a specificity of 79–100% (Table 1) (42–45). Recently published studies have also shown that 18-fluorodeoxyglucose [18F] FDG-PET/CT may efficiently identify even cranial GCA of the temporal arteries (43, 46–48).

A likely positive 18-FDG uptake is grade III, whereas a probable LVV is grade II.

It is critical to consider pre-analytical conditions that can affect 18-FDG uptake, such as hyperglycemia, tracer dose, and acquisition time between injections. Further, it is difficult to distinguish arteriosclerosis from LVV using 18-FDG uptake, but grade III uptake and involvement of the supra-aortic trunk or homogenous involvement of the entire aorta make it more likely to be due to vasculitis (3, 49–51).

By using the same interpretation modalities, the PETVAS score can help to homogenize interpretations and improve patient follow-up (52, 53).

The main limitations of FDG-PET/CT are linked to its inferior performance in cases of diabetes and its decreased sensitivity after commencing therapy with high doses of glucocorticoids. Three days of high-dose GC therapy can already attenuate FDG uptake of inflamed large vessels; such timeframe is still not defined for the assessment of temporal arteries with PET (54). In a prospective study, Imfeld et al. (55) evaluated the diagnostic performance of US and conventional [18F] FDG-PET/CT and concluded that both tests were complimentary. Indeed, typical [18F] FDG-PET/CT provides for greater exploration of the aorta, whereas ultrasonography allows for a better evaluation of cranial arteries (47, 48). When using PET/CT, one must consider the substantial irradiation of up to 25 mSv, making it not a standard imaging approach for diagnosing and monitoring of patients with GCA. Novel PET radiotracers that target cells (macrophages, T cells, and endothelial cells) implicated in the pathophysiology of GCA are being researched currently (56).

### 4.2. Magnetic resonance imaging and computed tomography angiography

Contrast MRI angiography (MRA) is used to examine cranial arteries, displaying arterial wall thickness and artery wall gadolinium enhancement. When compared to clinical diagnosis, a recent meta-analysis of ten studies of MRI in cranial-GCA revealed a pooled sensitivity and specificity of 75 and 89%, respectively. Sensitivity and specificity rose to 91 and 78%, respectively, when compared to TAB (Table 1) (57). Improved diagnostic performance for assessing wall thickness and mural enhancement in GCA patients was also established using fat-suppressed 3D High-resolution T1-weighted black-blood MRI (CUBE T1) versus 2D contrast-enhanced vessel-wall MRI (58). The benefit of adopting 3D MRI is its multiplanar reconstructions, which are beneficial when analyzing extracranial and intracranial arteries (59). Few studies have compared the accuracy of MRI to US in GCA patients. Yip et al. revealed that US was more sensitive than MRI in identifying changes in supra-aortic large arteries, particularly in individuals with chronic GCA (defined as active disease diagnosed at least 6 months before inclusion in the study). There were no variations in cranial artery evaluation between MRI and US (60). However, in a diagnostic emergency, MRI availability remains the fundamental barrier, while keeping in mind that this should not delay the delivery of glucocorticoids. A common method for diagnosing LVV is computed tomography angiography (CTA), which requires the intravenous administration of iodine-based contrast agents. After intravenous injection of a iodine-based contrast agent, arteritis on CTA manifests as mural thickening and double ring enhancement (61). In a prospective study of 24 patients with suspected GCA, 15 of whom were eventually diagnosed as GCA on an individual basis by experienced clinicians, mural thickening on CTA had a slightly lower specificity (84.6 versus 100%) and a positive predictive value (84.6 versus 100%) than increased FDG uptake on PET scanning, while sensitivity reached 73.3% for CTA and 66.7% for FDG-PET (42). In a study of 28 patients with GCA, de Boysson et al. (62) compared CTA to FDG-PET/CT. In a per-patient analysis, CTA demonstrated excellent sensitivity (95%) and specificity (100%) when compared to FDG-PET/CT. Sensitivity and specificity were 61 and 97.9%, respectively, in a per-segment analysis.

Few studies have found that CTA has high diagnostic accuracy. The authors of one study (42) observed a sensitivity of 73% and a specificity of 78% for the diagnosis of LV-GCA. Berthod et al. published a 2.2 mm aortic wall thickening threshold in favor of GCA (63). The primary limitation of CTA is the use of iodinated contrast material and irradiation, as well as the absence of evaluation of the temporal arteries.

## 5. Discussion

This mini-review focuses on the most updated evidence supporting the main tools available to diagnose and monitor LVV. The advantages, limitations, and innovative applications for each tool are discussed. The review highlights how the different diagnostic modalities should be used in a complementary way according to local availability and expertise, predominant clinical phenotype (cranial versus LV-GCA), timing from glucocorticoid treatment initiation (with the longest diagnostic yield demonstrated for TAB), patient’s preference, and cost considerations. Often, the different diagnostic or monitoring options can be applied in a step-wise fashion guided by pre-test clinical probability and initial findings (i.e., TAB requested in case of negative temporal artery US in a patient with predominantly cranial features, or PET/CT performed in a patient with negative axillary artery US and ongoing high suspicion for LV-GCA). One of the most relevant achievements emerging from the review is the increasing body of evidence supporting the role of imaging for the monitoring of the disease and to assess response to treatment which will considerably improve the management of GCA in the future.

## Author contributions

SM, VS, and FM contributed equally in the conception and writing of the manuscript. CS, CM, and RL contributed to the conception and design of the manuscript and critically revised it. All authors contributed to the article and approved the submitted version.

## References

[1] Schmidt W Kraft H Vorpahl K Völker L Gromnica-Ihle E. Color duplex ultrasonography in the diagnosis of temporal arteritis. *N Engl J Med.* (1997) 337:1336–42. 10.1056/NEJM199711063371902 9358127

[2] Chrysidis S Duftner C Dejaco C Schäfer V Ramiro S Carrara G Original article: definitions and reliability assessment of elementary ultrasound lesions in giant cell arteritis: a study from the OMERACT large vessel vasculitis ultrasound working group. *RMD Open.* (2018) 4:6098. 10.1136/rmdopen-2017-000598 29862043 PMC5976098

[3] Dejaco C Ramiro S Duftner C Besson F Bley T Blockmans D EULAR recommendations for the use of imaging in large vessel vasculitis in clinical practice. *Ann Rheum Dis.* (2018) 77:636–43. 10.1136/annrheumdis-2017-212649 29358285

[4] Luqmani R Lee E Singh S Gillett M Schmidt W Bradburn M The role of ultrasound compared to biopsy of temporal arteries in the diagnosis and treatment of giant cell arteritis (TABUL): a diagnostic accuracy and cost-effectiveness study. *Health Technol Assess Winch Engl.* (2016) 20:1–238. 10.3310/hta20900 27925577 PMC5165283

[5] Monti S Bartoletti A Bellis E Delvino P Montecucco C. Fast-track ultrasound clinic for the diagnosis of giant cell arteritis changes the prognosis of the disease but not the risk of future relapse. *Front Med.* (2020) 7:589794. 10.3389/fmed.2020.589794 33364248 PMC7753207

[6] Diamantopoulos A Haugeberg G Lindland A Myklebust G. The fast-track ultrasound clinic for early diagnosis of giant cell arteritis significantly reduces permanent visual impairment: towards a more effective strategy to improve clinical outcome in giant cell arteritis? *Rheumatol Oxf Engl.* (2016) 55:66–70. 10.1093/rheumatology/kev289 26286743

[7] Patil P Williams M Maw W Achilleos K Elsideeg S Dejaco C Fast track pathway reduces sight loss in giant cell arteritis: results of a longitudinal observational cohort study. *Clin Exp Rheumatol.* (2015) 33:103–6. 26016758

[8] Kermani T Warrington K Crowson C Hunder G Ytterberg S Gabriel S Predictors of dissection in aortic aneurysms from giant cell arteritis. *J Clin Rheumatol Pract Rep Rheum Musculoskelet Dis.* (2016) 22:184–7. 10.1097/RHU.0000000000000381 27219304

[9] Muratore F Kermani T Crowson C Green A Salvarani C Matteson E Large-vessel giant cell arteritis: a cohort study. *Rheumatol Oxf Engl.* (2015) 54:463–70. 10.1093/rheumatology/keu329 25193809 PMC4425829

[10] Ponte C Monti S Scirè C Delvino P Khmelinskii N Milanesi A Ultrasound halo sign as a potential monitoring tool for patients with giant cell arteritis: a prospective analysis. *Ann Rheum Dis.* (2021) 2:2021–220306. 34215646 10.1136/annrheumdis-2021-220306

[11] Stone J Tuckwell K Dimonaco S Klearman M Aringer M Blockmans D Trial of tocilizumab in giant-cell arteritis. *N Engl J Med.* (2017) 377:317–28. 10.1056/NEJMoa1613849 28745999

[12] Salvarani C Cantini F Boiardi L Hunder G. Polymyalgia rheumatica and giant-cell arteritis. *N Engl J Med.* (2002) 347:261–71. 10.1056/NEJMra011913 12140303

[13] Hellmich B Agueda A Monti S Buttgereit F de Boysson H Brouwer E 2018 update of the EULAR recommendations for the management of large vessel vasculitis. *Ann Rheum Dis.* (2019) 79:19–30. 10.1136/annrheumdis-2019-215672 31270110

[14] Maz M Chung S Abril A Langford C Gorelik M Guyatt G 2021 American college of rheumatology/vasculitis foundation guideline for the management of giant cell arteritis and takayasu arteritis. *Arthritis Rheumatol Hoboken NJ.* (2021) 73:1349–65. 10.1002/art.41774 34235884 PMC12344528

[15] Cavazza A Muratore F Boiardi L Restuccia G Pipitone N Pazzola G Inflamed temporal artery: histologic findings in 354 biopsies, with clinical correlations. *Am J Surg Pathol.* (2014) 38:1360–70. 10.1097/PAS.0000000000000244 25216320

[16] Restuccia G Cavazza A Boiardi L Pipitone N Macchioni P Bajocchi G Small-vessel vasculitis surrounding an uninflamed temporal artery and isolated vasa vasorum vasculitis of the temporal artery: two subsets of giant cell arteritis. *Arthr Rheum.* (2012) 64:549–56. 10.1002/art.33362 21953306

[17] Galli E Muratore F Boiardi L Restuccia G Cavazza A Catanoso M Significance of inflammation restricted to adventitial/periadventitial tissue on temporal artery biopsy. *Sem Arthr Rheum.* (2020) 50:1064–72. 10.1016/j.semarthrit.2020.05.021 32911285

[18] Rubenstein E Maldini C Gonzalez-Chiappe S Chevret S Mahr A. Sensitivity of temporal artery biopsy in the diagnosis of giant cell arteritis: a systematic literature review and meta-analysis. *Rheumatol Oxf Engl.* (2020) 59:1011–20. 10.1093/rheumatology/kez385 31529073

[19] Marvisi C Muratore F Cabassi C Galli E Boiardi L Piana S What to know about biopsy sampling and pathology in vasculitis? *Curr Rheumatol Rep.* (2022) 24:279–91. 10.1007/s11926-022-01082-6 35895226

[20] Mahr A Saba M Kambouchner M Polivka M Baudrimont M Brochériou I Temporal artery biopsy for diagnosing giant cell arteritis: the longer, the better? *Ann Rheum Dis.* (2006) 65:826–8. 10.1136/ard.2005.042770 16699053 PMC1798165

[21] Muratore F Boiardi L Cavazza A Tiengo G Galli E Aldigeri R Association between specimen length and number of sections and diagnostic yield of temporal artery biopsy for giant cell arteritis. *Arthr Care Res.* (2021) 73:402–8. 10.1002/acr.24393 32741116

[22] Chakrabarty A Franks A. Temporal artery biopsy: is there any value in examining biopsies at multiple levels? *J Clin Pathol.* (2000) 53:131–6. 10.1136/jcp.53.2.131 10767829 PMC1763285

[23] Niederkohr R Levin L. Management of the patient with suspected temporal arteritis a decision-analytic approach. *Ophthalmology.* (2005) 112:744–56. 10.1016/j.ophtha.2005.01.031 15878052

[24] Achkar A Lie J Hunder G O’Fallon W Gabriel S. How does previous corticosteroid treatment affect the biopsy findings in giant cell (temporal) arteritis? *Ann Intern Med.* (1994) 120:987–92. 10.7326/0003-4819-120-12-199406150-00003 8185147

[25] Narváez J Bernad B Roig-Vilaseca D García-Gómez C Gómez-Vaquero C Juanola X Influence of previous corticosteroid therapy on temporal artery biopsy yield in giant cell arteritis. *Sem Arthr Rheum.* (2007) 37:13–9. 10.1016/j.semarthrit.2006.12.005 17360027

[26] Bury D Joseph J Dawson T. Does preoperative steroid treatment affect the histology in giant cell (cranial) arteritis? *J Clin Pathol.* (2012) 65:1138–40. 10.1136/jclinpath-2012-200870 22844066

[27] Maleszewski J Younge B Fritzlen J Hunder G Goronzy J Warrington K Clinical and pathological evolution of giant cell arteritis: a prospective study of follow-up temporal artery biopsies in 40 treated patients. *Mod Pathol Off J U S Can Acad Pathol Inc.* (2017) 30:788–96. 10.1038/modpathol.2017.10 28256573 PMC5650068

[28] Visvanathan S Rahman M Hoffman G Xu S García-Martínez A Segarra M Tissue and serum markers of inflammation during the follow-up of patients with giant-cell arteritis–a prospective longitudinal study. *Rheumatol Oxf Engl.* (2011) 50:2061–70. 10.1093/rheumatology/ker163 21873264 PMC3198905

[29] Germanò G Muratore F Cimino L Lo Gullo A Possemato N Macchioni P Is colour duplex sonography-guided temporal artery biopsy useful in the diagnosis of giant cell arteritis? A randomized study. *Rheumatol Oxf Engl.* (2015) 54:400–4. 10.1093/rheumatology/keu241 24939678

[30] Duftner C Dejaco C Sepriano A Falzon L Schmidt W Ramiro S. Imaging in diagnosis, outcome prediction and monitoring of large vessel vasculitis: a systematic literature review and meta-analysis informing the EULAR recommendations. *RMD Open.* (2018) 4:e000612. 10.1136/rmdopen-2017-000612 29531788 PMC5845406

[31] Sebastian A Coath F Innes S Jackson J van der Geest K Dasgupta B. Role of the halo sign in the assessment of giant cell arteritis: a systematic review and meta-analysis. *Rheumatol Adv Pract.* (2021) 5:rkab059. 10.1093/rap/rkab059 34514295 PMC8421813

[32] Monti S Floris A Ponte C Schmidt W Diamantopoulos A Pereira C The use of ultrasound to assess giant cell arteritis: review of the current evidence and practical guide for the rheumatologist. *Rheumatol Oxf Engl.* (2017) 57:227–35. 10.1093/rheumatology/kex173 28460064 PMC5850748

[33] Skoog J Svensson C Eriksson P Sjöwall C Zachrisson H. The diagnostic performance of an extended ultrasound protocol in patients with clinically suspected giant cell arteritis. *Front Med.* (2021) 8:807996. 10.3389/fmed.2021.807996 35118098 PMC8804250

[34] Ješe R Rotar Ž Tomšič M Hočevar A. The cut-off values for the intima-media complex thickness assessed by colour doppler sonography in seven cranial and aortic arch arteries. *Rheumatol Oxf Engl.* (2021) 60:1346–52. 10.1093/rheumatology/keaa578 32944770

[35] Schäfer V Juche A Ramiro S Krause A Schmidt W. Ultrasound cut-off values for intima-media thickness of temporal, facial and axillary arteries in giant cell arteritis. *Rheumatol Oxf Engl.* (2017) 56:1479–83. 10.1093/rheumatology/kex143 28431106

[36] Monti S Ponte C Pereira C Manzoni F Klersy C Rumi F The impact of disease extent and severity detected by quantitative ultrasound analysis in the diagnosis and outcome of giant cell arteritis. *Rheumatol Oxf Engl.* (2020) 59:2299–307. 10.1093/rheumatology/kez554 31848610

[37] van der Geest K Borg F Kayani A Paap D Gondo P Schmidt W Novel ultrasonographic halo score for giant cell arteritis: assessment of diagnostic accuracy and association with ocular ischaemia. *Ann Rheum Dis.* (2020) 79:393–9. 10.1136/annrheumdis-2019-216343 31900304 PMC7034352

[38] Ponte C Serafim A Monti S Fernandes E Lee E Singh S Early variation of ultrasound halo sign with treatment and relation with clinical features in patients with giant cell arteritis. *Rheumatol Oxf Engl.* (2020) 59:3717–26. 10.1093/rheumatology/keaa196 32393983

[39] Seitz L Christ L Lötscher F Scholz G Sarbu A Bütikofer L Quantitative ultrasound to monitor the vascular response to tocilizumab in giant cell arteritis. *Rheumatol Oxf Engl.* (2021) 60:5052–9. 10.1093/rheumatology/keab484 34117737 PMC8566271

[40] Schäfer V Chrysidis S Schmidt W Duftner C Iagnocco A Bruyn G OMERACT definition and reliability assessment of chronic ultrasound lesions of the axillary artery in giant cell arteritis. *Semin Arthr Rheum.* (2021) 51:951–6. 10.1016/j.semarthrit.2021.04.014 34140184

[41] Bosch P Dejaco C Schmidt W Schlüter K Pregartner G Schäfer V. Ultrasound for diagnosis and follow-up of chronic axillary vasculitis in patients with long-standing giant cell arteritis. *Ther Adv Musculoskelet Dis.* (2021) 13:505. 10.1177/1759720X21998505 33796156 PMC7983430

[42] Lariviere D Benali K Coustet B Pasi N Hyafil F Klein I Positron emission tomography and computed tomography angiography for the diagnosis of giant cell arteritis: a real-life prospective study. *Medicine.* (2016) 95:e4146. 10.1097/MD.0000000000004146 27472684 PMC5265821

[43] Thibault T Durand-Bailloud B Soudry-Faure A Greigert H Drouet C Devilliers H PET/CT of cranial arteries for a sensitive diagnosis of giant cell arteritis. *Rheumatology.* (2022) 22:430. 10.1093/rheumatology/keac430 35866984

[44] Hay B Mariano-Goulart D Bourdon A Benkiran M Vauchot F De Verbizier D Diagnostic performance of 18F-FDG PET-CT for large vessel involvement assessment in patients with suspected giant cell arteritis and negative temporal artery biopsy. *Ann Nucl Med.* (2019) 33:512–20. 10.1007/s12149-019-01358-5 30976984

[45] Prieto-González S Depetris M García-Martínez A Espígol-Frigolé G Tavera-Bahillo I Corbera-Bellata M Positron emission tomography assessment of large vessel inflammation in patients with newly diagnosed, biopsy-proven giant cell arteritis: a prospective, case-control study. *Ann Rheum Dis.* (2014) 73:1388–92. 10.1136/annrheumdis-2013-204572 24665112

[46] Sammel A Hsiao E Schrieber L Janssen B Youssef P Fraser C Fluorine-18 fluoro-2-deoxyglucose positron emission tomography uptake in the superficial temporal and vertebral arteries in biopsy positive giant cell arteritis. *J Clin Rheumatol Pract Rep Rheum Musculoskelet Dis.* (2017) 23:443. 10.1097/RHU.0000000000000543 28953579

[47] Nielsen B Hansen I Kramer S Haraldsen A Hjorthaug K Bogsrud T Simple dichotomous assessment of cranial artery inflammation by conventional 18F-FDG PET/CT shows high accuracy for the diagnosis of giant cell arteritis: a case-control study. *Eur J Nucl Med Mol Imaging.* (2019) 46:184–93. 10.1007/s00259-018-4106-0 30066157

[48] Sammel A Hsiao E Schembri G Nguyen K Brewer J Schrieber L Diagnostic accuracy of positron emission tomography/computed tomography of the head, neck, and chest for giant cell arteritis: a prospective, double-blind, cross-sectional study. *Arthr Rheumatol.* (2019) 71:1319–28. 10.1002/art.40864 30848549

[49] Rhja S. FDG-PET/CT(A) imaging in large vessel vasculitis and polymyalgia rheumatica: joint procedural recommendation of the EANM, SNMMI, and the PET interest group (PIG), and endorsed by the ASNC. *Eur J Nucl Med Mol Imaging.* (2018) 45:29637252. 10.1007/s00259-018-3973-8 29637252 PMC5954002

[50] Espitia O Schanus J Agard C Kraeber-Bodéré F Hersant J Serfaty J Specific features to differentiate Giant cell arteritis aortitis from aortic atheroma using FDG-PET/CT. *Sci Rep.* (2021) 11:34462502. 10.1038/s41598-021-96923-2 34462502 PMC8405613

[51] Rosenblum J Quinn K Rimland C Mehta N Ahlman M Grayson P. Clinical factors associated with time-specific distribution of 18f-fluorodeoxyglucose in large-vessel vasculitis. *Sci Rep.* (2019) 9:31645635. 10.1038/s41598-019-51800-x 31645635 PMC6811531

[52] Kang F Han Q Zhou X Zheng Z Wang S Ma W Performance of the PET vascular activity score (PETVAS) for qualitative and quantitative assessment of inflammatory activity in Takayasu’s arteritis patients. *Eur J Nucl Med Mol Imaging.* (2020) 47:3107–17. 10.1007/s00259-020-04871-2 32567005

[53] Alessi H Quinn K Ahlman M Novakovich E Saboury B Luo Y Longitudinal characterization of vascular inflammation and disease activity in takayasu arteritis and giant cell arteritis: a single-center prospective study. *Arthritis Care Res.* (2022). [Epub ahead of print]. 10.1002/acr.24976 35762866 PMC12189024

[54] Nielsen B Gormsen L Hansen I Keller K Therkildsen P Hauge E. Three days of high-dose glucocorticoid treatment attenuates large-vessel 18F-FDG uptake in large-vessel giant cell arteritis but with a limited impact on diagnostic accuracy. *Eur J Nucl Med Mol Imaging.* (2018) 45:1119–28. 10.1007/s00259-018-4021-4 29671039

[55] Imfeld S Aschwanden M Rottenburger C Schegk E Berger C Staub D [18F]FDG positron emission tomography and ultrasound in the diagnosis of giant cell arteritis: congruent or complementary imaging methods? *Rheumatology.* (2020) 59:772–8. 10.1093/rheumatology/kez362 31436837

[56] van der Geest K Sandovici M Nienhuis P Slart R Heeringa P Brouwer E Novel PET imaging of inflammatory targets and cells for the diagnosis and monitoring of giant cell arteritis and polymyalgia rheumatica. *Front Med.* (2022) 9:902155. 10.3389/fmed.2022.902155 35733858 PMC9207253

[57] Zhang K Li M Zhang P Qin H Guo Z Yang Y. Validity of high resolution magnetic resonance imaging in detecting giant cell arteritis: a meta-analysis. *Eur Radiol.* (2022) 32:3541–52. 10.1007/s00330-021-08413-8 35015125

[58] Rodriguez-Régent C Ben Hassen W Seners P Oppenheim C Régent A. 3D T1-weighted black-blood magnetic resonance imaging for the diagnosis of giant cell arteritis. *Clin Exp Rheumatol.* (2020) 124:95–8. 32301421

[59] Poillon G Collin A Benhamou Y Clavel G Savatovsky J Pinson C Increased diagnostic accuracy of giant cell arteritis using three-dimensional fat-saturated contrast-enhanced vessel-wall magnetic resonance imaging at 3 T. *Eur Radiol.* (2020) 30:31811430. 10.1007/s00330-019-06536-7 31811430

[60] Yip A Jernberg E Bardi M Geiger J Lohne F Schmidt W Magnetic resonance imaging compared to ultrasonography in giant cell arteritis: a cross-sectional study. *Arthritis Res Ther.* (2020) 22:247. 10.1186/s13075-020-02335-4 33076985 PMC7574248

[61] Schmidt W Nielsen B. Imaging in large-vessel vasculitis. *Best Pract Res Clin Rheumatol.* (2020) 34:101589. 10.1016/j.berh.2020.101589 32948434

[62] de Boysson H Dumont A Liozon E Lambert M Boutemy J Maigné G Giant-cell arteritis: concordance study between aortic CT angiography and FDG-PET/CT in detection of large-vessel involvement. *Eur J Nucl Med Mol Imaging.* (2017) 44:2274–9. 10.1007/s00259-017-3774-5 28736805

[63] Berthod P Aho-Glélé S Ornetti P Chevallier O Devilliers H Ricolfi F CT analysis of the aorta in giant-cell arteritis: a case-control study. *Eur Radiol.* (2018) 28:3676–84. 10.1007/s00330-018-5311-8 29600479

[64] Klink T Geiger J Both M Ness T Heinzelmann S Reinhard M Giant cell arteritis: diagnostic accuracy of MR imaging of superficial cranial arteries in initial diagnosis-results from a multicenter trial. *Radiology.* (2014) 273:844–52. 10.1148/radiol.14140056 25102371

[65] Quinn K Dashora H Novakovich E Ahlman M Grayson P. Use of 18F-fluorodeoxyglucose positron emission tomography to monitor tocilizumab effect on vascular inflammation in giant cell arteritis. *Rheumatol Oxf Engl.* (2021) 60:4384–9. 10.1093/rheumatology/keaa894 33369678 PMC8633659

